# A specialist-generalist classification of the arable flora and its response to changes in agricultural practices

**DOI:** 10.1186/1472-6785-10-20

**Published:** 2010-09-01

**Authors:** Guillaume Fried, Sandrine Petit, Xavier Reboud

**Affiliations:** 1 INRA, UMR1210 Biologie et Gestion des Adventices, F-21000 Dijon, France; 2 LNPV, Station d'Entomologie et Plantes Invasives, F-34000 Montpellier, France

## Abstract

**Background:**

Theory in ecology points out the potential link between the degree of specialisation of organisms and their responses to disturbances and suggests that this could be a key element for understanding the assembly of communities. We evaluated this question for the arable weed flora as this group has scarcely been the focus of ecological studies so far and because weeds are restricted to habitats characterised by very high degrees of disturbance. As such, weeds offer a case study to ask how specialization relates to abundance and distribution of species in relation to the varying disturbance regimes occurring in arable crops.

**Results:**

We used data derived from an extensive national monitoring network of approximately 700 arable fields scattered across France to quantify the degree of specialisation of 152 weed species using six different ecological methods. We then explored the impact of the level of disturbance occurring in arable fields by comparing the degree of specialisation of weed communities in contrasting field situations.

The classification of species as specialist or generalist was consistent between different ecological indices. When applied on a large-scale data set across France, this classification highlighted that monoculture harbour significantly more specialists than crop rotations, suggesting that crop rotation increases abundance of generalist species rather than sets of species that are each specialised to the individual crop types grown in the rotation. Applied to a diachronic dataset, the classification also shows that the proportion of specialist weed species has significantly decreased in cultivated fields over the last 30 years which suggests a biotic homogenization of agricultural landscapes.

**Conclusions:**

This study shows that the concept of generalist/specialist species is particularly relevant to understand the effect of anthropogenic disturbances on the evolution of plant community composition and that ecological theories developed in stable environments are valid in highly disturbed environments such as agro-ecosystems. The approach developed here to classify arable weeds according to the breadth of their ecological niche is robust and applicable to a wide range of organisms. It is also sensitive to disturbance regime and we show here that recent changes in agricultural practices, i.e. increased levels of disturbance have favoured the most generalist species, hence leading to biotic homogenisation in arable landscapes.

## Background

The concept of ecological niche as a hypothetical multidimensional space [1] has boosted the exploration of niche properties [2-4] and has enabled generalist and specialist species to be distinguished according to their respective niche breadth. Theoretical studies have tried to evaluate the origins and/or the consequences of niche-breadth differences among species. The "jack-of-all-trades is master of none" hypothesis states that the existence of generalist versus specialist species is the result of an evolutionary trade-off between the ability of species to use an extended range of resources and their capacity to exploit each one with a level of performance above those of competing species [5-8]. This trade-off has been associated with several life-history traits: generalist species are supposed to maintain higher dispersal abilities [9,10] and to cope more easily with environmental stochasticity [11] while, conversely, specialists would be strongly shaped by intra-specific competition [12]. The generalist-specialist concept could thus be appropriate to find community assembly rules [13], in particular in habitats where communities are subjected to varying levels of disturbance. The distinction between generalist and specialist species can pinpoint general mechanisms of species filtering, similarly to approaches using species traits rather than the species themselves. Indeed, several authors have recently focused on the processes leading to the replacement of many specialist species by a few generalist species that take place in diverse phylogenetic groups such as fish [14], bird [15] or plant assemblages [16]. In parallel to species extinction, this so called 'biotic homogenization' process would characterize the next biodiversity crisis [17].

The weed communities of arable land provide an interesting model for exploring the generalist/specialist concept because the arable field habitat is characterized by an intense disturbance regime and by varying ecological conditions, both within a year (because of management practices) and across years (because of crop rotation). Compared to other vascular plants occurring in more stable habitats, one would expect arable weeds to be generalist species. Indeed, it has been hypothesized that "under a constant environment or slow environmental changes, inter-specific competition involves local processes that favour specialist species at the expense of generalist species, while under moderate to high rates of environmental change, local population dynamics increasingly favour high immigration rates of the generalist over the local competitive ability of the specialist" [18]. However, arable fields can also be viewed as a particular habitat harbouring a set of specialized species (i.e. arable weeds) adapted to frequent but also specific disturbances. Within a year, agricultural practices can be perceived as highly specialized with the single aim of favouring a particular species, i.e. the crop, so that all species in the seed bank sharing the same requirements might well find regularly optimal conditions for completing their life cycle. Large differences between habitat breadth of weed species are observed with some species confined to arable fields in Western Europe (e.g., weeds of winter cereal fields: *Agrostemma githago, Bupleurum rotundifolium*, etc.) while others are able to grow both in crops and in other less disturbed habitats (e.g., *Galium aparine, Lapsana communis*). Even within arable fields, differences can be observed in niche position and breadth between weeds that are specialized to a particular crop type or to particular soil conditions, and weeds that are present almost everywhere [19,20]. Finally, even if arable fields are characterized by stochastic conditions, a specialized species could persist temporally, for example, with dormant diaspores that would wait for optimal environmental conditions e.g., high relative summer air humidity for arable bryophyte species [21] or each time a favourable crop occurs in the rotation [22]. Therefore, even though most agroecosystems experience high levels of disturbance, it is not clear whether the current arable weed flora is dominated by specialist or by generalist species and what would be the resulting variation in ecosystem function [23]. The present paper addresses this question and classifies the French arable weed flora along a specialist-generalist gradient, using vegetation records from a national monitoring network for applying and comparing six different specialisation indices available in the ecological literature. It should be noted here that what is meant by generalist species are species able to exploit many or all the niches within the "arable field" habitat regardless of their ability to occupy other habitats.

A second set of questions relates to possible relationships between the degree of specialisation of weed communities and disturbance regime that are related to the choice of contrasted agricultural management options. In this paper, we focus on two sets of situations that result in different levels and regime of disturbance.

The first situation compares the weed flora in maize cultivated as a monoculture and in a crop rotation. Monoculture means here that the same crop species is cultivated for several consecutive years. Crop rotation means that each year a different crop species is cultivated. Crop rotation induces disturbances that vary with the crop grown each year (planting or maturation dates, growth habit, competitive ability, associated cultural practices, fertiliser requirements and more or less specific herbicides) while the disturbance regime is constant in monocultures. While many recent studies focused on the impact of crop rotation on weed diversity [24-26], the present study aimed to quantify the functional shift in weed composition. There could be two alternative responses of weed communities to crop rotation (i) crop rotation could either favour generalist species and monoculture specialist species, or, (ii) crop rotation could favour specialist species of each crop (i.e., species that are associated with conditions of a particular crop), so that weed communities would mainly be composed of specialist species alternating each year, persisting within the seed bank during the unfavourable years.

The second situation analyses the shift in weed communities that has taken place in the same arable fields between the 1970 s and the 2000 s. It is here assumed that the level of disturbance has significantly increased with agriculture intensification (i.e., increasing number of herbicide treatments, increasing depth and frequency of tillage, see [27]) between the two surveys. Increased N-fertilization and systematic liming or drainage have homogenized soil conditions across the sampled fields. In addition, recurrent changes in cultivation techniques since the 1970 s (tillage or no-tillage systems, new herbicides) are likely to have translated into continuous environmental changes for the arable flora. These changes may have hampered specialist species and/or favoured generalist weed species.

## Results

### Classification of weed species along the gradient of specialization

**Figure 1:**
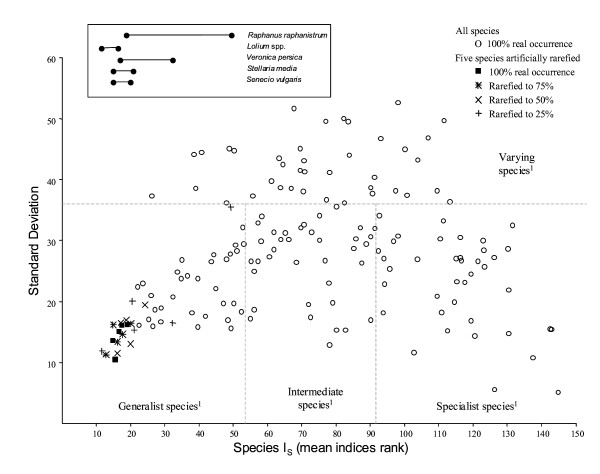
**Weed species mean rank and standard deviation according to the six methods of classification along a specialist/generalist index**. The mean rank and standard deviation of all weed species and for the 5 weed species artificially rarefied. The Y-axis gives the standard deviation of the mean value according to the 6 indices. The box gives the range of values for the five species. The full lists of the most generalist, intermediate, specialist and varying species are given in the Electronic Supplementary Material.

**Figure 2:**
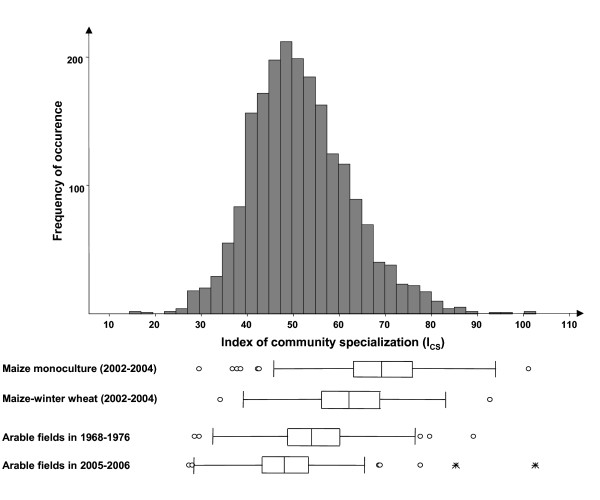
**Distribution of the values of the average degree of specialization (I_CS_) of arable weed communities**. Box plots represent the comparison between the I_CS _of weed communities of the 1970 s and of the 2000 s (n = 158 fields) and between maize cropped as a monoculture (n = 235 fields) or within a two-year maize/winter wheat crop rotation (n = 169 fields). Boxes represent interquartile range, containing 50% of values; the line across boxes is the median values; the whiskers are drawn from the top of the box up to the largest data point less than 1.5 times the box height from the box (the "upper inner fence"), and similarly below the box, outlying values shown as circles, values more than 3 times the box height from the box (the "outer fences") are shown as stars.

In total, 152 weed species were frequent enough to estimate their degree of specialisation (I_S_) with six different indices (See additional file 1: Classification of arable weed species according to their niche breadth). Each pair of indices were significantly correlated (Table 1). The I_S _values varied from 15 for *Stellaria media*, the most generalist species, to 145 for *Arenaria serpyllifolia*, the most specialist species (Fig. 1). The segregation of the values of I_S _in three classes of equal size (n = 38 species) enabled us to distinguish the most generalist species, from I_S _= 15 to I_S _= 53, intermediate species from I_S _= 54 to I_S _= 92 and the most specialist species, from I_S _= 93 to I_S _= 145. At the level of communities, the I_CS _values (i.e., the mean I_S _of the species present in the community) follow a normal distribution and varied from 14.33 to 102.58 (Fig. 2).

**Table 1:** Spearman's rank correlation test between the six indices of species niche breadth.

	I1 (RS)	I2 (CCA-SD)	I3 (CCA-Rao)	I4(OMI)	I5 (IV)	I6 (Sophy)
**I1(RS)**	1	0.615**	0,736**	0,315**	0,182*	0,692**
**I2(CCA-SD)**		1	0.863**	0.602**	0.309**	0.530**
**I3(CCA-Rao)**			1	0.475**	0.210**	0.630**
**I4(OMI)**				1	0.260**	0.226**
**I5(IV)**					1	0.153*
**I6(Sophy)**						1

Abbreviations: see Table 1. *P < 0.05; ** *P *< 0.01

*Note: *correlations are based on n = 152 weed species

**Figure 3:**
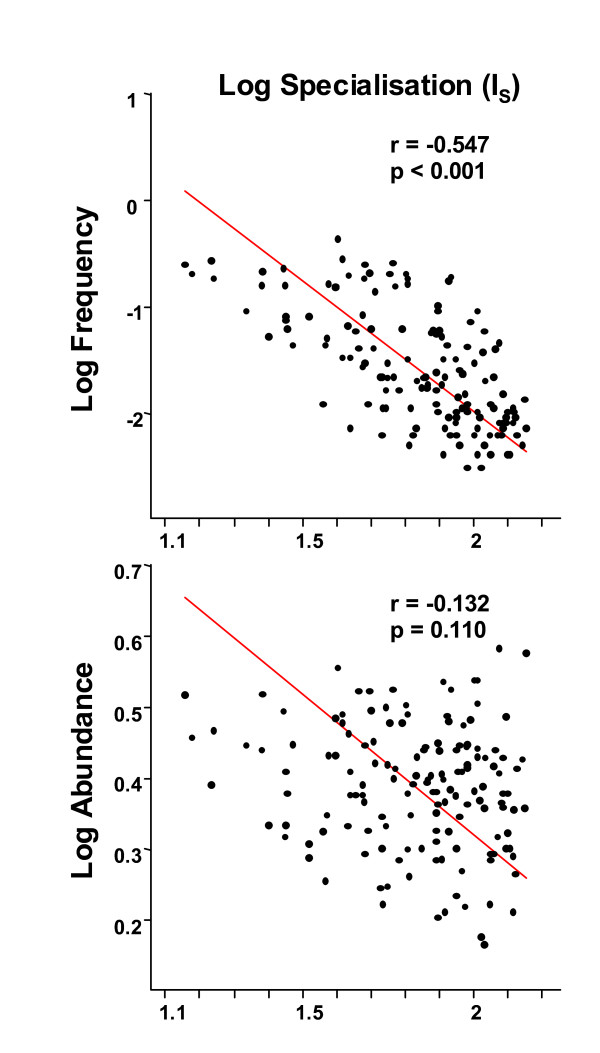
**Relationships between weed species specialisation and (a) species frequency and (b) species local abundance**. *Note: *correlations are based on n = 152 plant species.

I_S _was negatively correlated with the frequency of occurrence of the species (r= -0.547; P < 0.001) but not significantly with their abundance (r = -0.132; P = 0.110) (Fig. 3). With the exception of *Raphanus raphanistrum *at 25% of its actual frequency, all six indices classified the species as generalists with very similar positions regardless of their rarefied frequency (Fig. 1).

The most generalist species (e.g. *Stellaria media*, *Senecio vulgaris*, *Capsella bursa-pastoris*, *Anagallis arvensis*) were found on a large range of soil types, climates, crop types and cropping techniques. In fact these species are found all over Western Europe [28], and even in other temperate countries such as Canada. At the opposite extreme, the most specialist species were weed species that all seemed highly specialized on at least two important axes of their n-dimensional niche, e.g., *Rumex acetosella *was only found in winter crops exhibiting very acidic and sandy soil conditions, while *Phalaris paradoxa *was only observed under rather oceanic climate, heavy clay and wet soil conditions. At an intermediary level, some species were specialized on one important axis of their niche while being present on a broader range of ecological conditions on other axes. For example, *Papaver rhoeas *or *Veronica hederifolia *were associated with a particular crop type (winter cereals) but were present on a large range of soil and climatic conditions while *Juncus bufonius *was strictly limited to acid and silty soils but was found in various crop types.

### Effect of monoculture versus crop rotation

**Figure 4:**
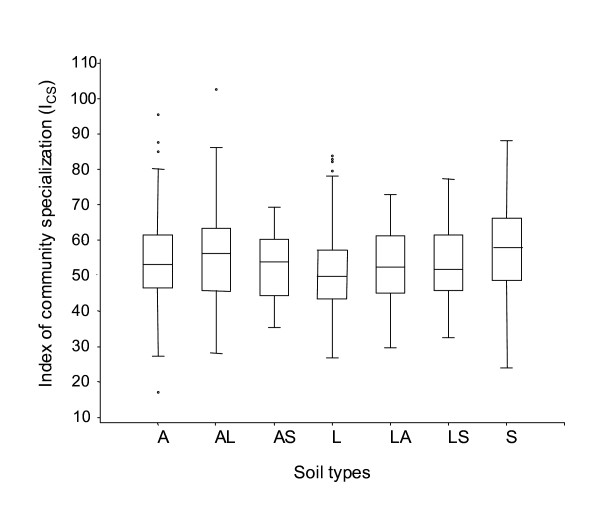
**Distribution of I_CS _values in maize fields according to soil types**. *Note*: n = 404 fields i.e. 235 cropped as monoculture and 169 as crop rotation; A: clay; AL: clay loam; AS: sandy clay; L: silt loam; LA: silty clay; LS: sandy loam; S: sand.

The comparison of maize weed communities in crop rotation *versus *monoculture showed similar field species richness or abundance (Table 2) while the average specialization index (I_CS_) indicated different species composition (Fig. 2). More generalist species were observed in fields with maize-wheat rotations (I_CS _= 62 +/- 2), whereas maize monocultures contained more specialist species (I_CS _= 69 +/-1, Wilcoxon test, *P *< 0.01). This was mainly due to the high proportion of generalist species when maize is grown in crop rotation (Table 2). The generalist *Chenopodium album *was the dominant species in both monoculture and crop rotation, followed by the two specialist species *Echinochloa crus-galli *and *Amaranthus retroflexus *in monocultures and the two generalist species *Polygonum aviculare *and *Fallopia convolvulus *in rotations. The two cropping systems were not evenly distributed between the different soil types, with proportionately more fields with crop rotation on clay soils and sandy clay soils, and more fields with monocultures on sandy soils (Table 3). However, the distribution of I_CS _values across soil types presented in Fig. 4 shows no significant effect of soil type on I_CS _of weed communities.

**Table 2:** Mean species richness, abundance and ecological specialization of weed communities per field:

	**a - **Between cropping systems			**b **- Over time		
	
	Mo	Ro	P	1970s	2000s	P
*Wilcoxon Test*						

Mean species richness	12.59	14.06	P = 0.07	16.56	9.34	P < 0.01
Mean abundance	9.56	8.11	P = 0.07	61.5	20.2	P < 0.01

*Khi2 Test*	*Khi2 = 84.5*		*P < 0.01*	*Khi2 = 64.0*		*P < 0.01*

Sum of occurrences for:						
*Generalist species*	1129	1154		2180	1351	
*Intermediate species*	777	485		676	405	
*Specialist species*	429	268		344	99	
Total	2959	2377		3200	1885	

**a- **in maize fields cultivated as monoculture (Mo, n = 235) and in maize-wheat rotations (Ro, n = 169) in the 2000 s,

**b- **for the same fields over time between the 1970 s and the 2000 s (n = 158 fields).

**Table 3:** Distribution of cropping system according to soil types.

Soil types (texture)	Monoculture	Crop rotation	Total
Clay	16	15	31
Clay loam	36	21	57
Sandy clay	7	9	16
Silt loam	25	13	38
Silty clay	12	10	22
Sandy loam	9	7	16
Sand	44	4	48

Total	150	78	228

Khi2 Test, Khi2 = 22.4, P < 0.005

### Evolution of the specialist-generalist ratio over the last 30 years

Between the 1970 s and the 2000 s, weed species richness significantly decreased from 16.56 to 9.34 species per field and species density from 61.5 individuals per m^2 ^to 20.2 (Table 2). Among the 121 species recorded in both surveys, 48 species significantly decreased in frequency and only 12 significantly increased.

The index of community specialization I_CS _significantly decreased from I_CS _= 54.25 +/- 0.97 in the 1970 s to I_CS _= 49.02 +/- 1.04 in the 2000 s (Fig. 2, Wilcoxon-Test, P < 0.001). The frequency of generalist species was either stable (*Capsella bursa-pastoris, Lolium multiflorum, Poa annua, Taraxacum officinale*) or increased (*Lactuca serriola, Senecio vulgaris*), while the frequency of specialist species fell (*Arenaria serpyllifolia, Lithospermum arvense, Legousia speculum-veneris, Stachys arvensis*). There was a higher proportion of specialist species in the 1970 s while the weed communities of the 2000 s were characterized by a higher proportion of intermediate and generalist species, with 90% of the effect attributable to the loss of specialist species over time (Table 2).

## Discussion

### Robustness of the specialization index

The use of several indices was not to assess the performance and relative value of the different methods but rather to increase the robustness of the results. The six indices representing different measures of species niche breadth gave consistent and correlated classifications of species, from the most specialized to the most ubiquitous. Not surprisingly, there were exceptions, notably *Plantago lanceolata*, ranked according to I2 as the most generalist species while the other five indices consistently classified this species as intermediate. Such differences presumably arose when species are regarded as generalist or not according to which niche axes were included in the analysis (e.g. soil, climate or crop type).

The significant correlations between the pairs of indices seem to indicate that the choice of an index based on species co-occurrence or on precise environmental data with different ordination or classification methods does not alter the ranking for most of the species. It is worth noting that indices based solely on species co-occurrence such as I6 demonstrated that increasingly available, large-survey datasets could yield information on species niche-breadth without detailed environmental or habitat measurements [29].

### Frequency and abundance of species according to their niche breadth

The species with the largest niche breadth were also the most frequent but not necessarily the most abundant (Fig. 3). The random rarefaction of five common species in the dataset did not alter the classification of these species as generalists which tends to indicate that I_S _is not directly affected by the number of samples where the species concerned occurs (Fig. 1). This result partially supports the 'resource breadth hypothesis' [30] that states that 'species with broad environmental tolerance and able to use a wide variety of resources ('generalist species') would survive in more places and over larger areas'. It is however not clear to what extent the relationship could result from a sampling artefact, i.e. when data has been collected along a large environmental gradient, very frequent species will also appear as generalist species [31]. However, the reverse is not necessarily true: a rare species could either be generalist (if the rare sites where they occur are very different) or specialist (if all the sites where they occur have close ecological conditions and sufficient connectivity to be occupied). The absence of any strong relationships between niche breadth and species mean abundance means however that, contrary to the 'resource breadth hypothesis', not all generalist weed species would achieve high local densities. Moreover, the reverse seems often true in cultivated fields, since arable weeds that appear as specialists of a crop (for example *Digitaria sanguinalis *or *Setaria pumila *due to herbicide resistance in maize fields) can form very dense populations.

### The effect of disturbance dynamics on the assembly of communities

We show here that a two-year cycle combining maize and winter wheat enhances the representation of generalist species in the weed communities found in maize (48% of species) compared to maize cultivated as monoculture (37%). Maize grown every two to three years does not appear to be sufficient in terms of (geometric) fitness advantage for specialist weed species to outperform the generalist species that are able to cope with alternating crops. Our results also suggest that in maize grown in a rotation, the annual change of crop sowing dates and associated practices have caused a shift in the weed flora in favour of 'germination generalist', i.e. species that can germinate all-year-round or at least in both autumn and spring (*Polygonum aviculare*, *Lolium multiflorum*, *Alopecurus myosuroides*, *Anagallis arvensis*, *Galium aparine*, *Fumaria officinalis*, *Cirsium arvense *and *Viola arvensis*) [32]. The annual changes in herbicide selectivity limit the selection of specialist species associated to a particular crop [33]. In the monoculture situation, although many generalist species were still observed, the repetition of a similar selection pressure each year appears to have favoured some of the efficient and well adapted species such as the intermediary-specialized *Calystegia sepium*, *Cynodon dactylon*, *Amaranthus retroflexus *or the specialized *Digitaria sanguinalis*, *Datura stramonium *or *Setaria pumila *species. It is worth noting that we found no significant differences in either weed diversity or weed density between crop rotation and monoculture which although counter-intuitive [24] is in line with several studies showing that the effect varies according to the rotation that is being considered [34,35]. As in the case of the present study, it is possible that the ratio of specialist-generalist species in weed communities proves to be a more responsive indicator of the effect of monoculture versus crop rotation. This remains to be explored in further studies.

### Longer-term agricultural changes and the increasing proportion of generalist species

The decline of arable weeds during the last decades has been reported in various European countries [36-38]. In this paper, we show that in addition to the loss in the number of species, the specialist-generalist ratio in weed communities has significantly changed over the last decades. This trend is mostly the result of a more pronounced decrease in the occurrence of specialist species, while during the same period intermediate and generalist species remained more often stable or even increased. For example, *Legousia speculum-veneris *and *Lithospermum arvense *are two specialist weeds of winter cereals on calcareous soils that are in decline most probably due to their sensitivity to the main herbicides used in cereals. The decline of *Gnaphalium uliginosum*, *Misopates orontium *and *Stachys arvensis*, specialist species of acidic and sandy soils, could be related to the agricultural practice of liming. Species of highly drained soils with poor competing capacity (like the specialist weed *Arenaria serpyllifolia*) could have been eliminated by an increased level of fertilization and the resulting increased competition with other plants, mostly the crop itself. On the other hand, the increase or the maintenance of species such as *Senecio vulgaris*, *Matricaria perforata*, *Cirsium arvense*, *Poa annua *or *Lolium spp*. can be explained in part by their generalist properties i.e. i) the capacity to germinate all year round and thus the possibility that some cohorts avoid herbicides pressures or periods of intense competition with the crop species, ii) the lack of specialisation to individual crop types and thus the lack of response to shifts in the acreage devoted to specific crops and iii) the tolerance to a broad range of soil types and thus the lack of response to changes in agricultural practices modifying ecological conditions (fertilization, liming, drainage, irrigation). Our results therefore indicate that not only did agro-ecosystems lose a significant number of weed species in the recent decades, but those that remain also are the most generalist, which could lead to a decreased differentiation of weed communities found in different crop types in the longer term. This confirms that the biotic homogenisation process reported in other plant groups in rural landscapes [16] also affects weed communities found in cultivated fields.

There may be an apparent contradiction in the results between the selection of generalists over the last 30 years of agricultural intensification and the selection of specialists by monocultures (which is also perceived as part of the intensification process). However, this paradox disappears when analyzing the nature of the species specialization in each situation. In the diachronic long term study, the ratio of generalist/specialist increases because a lot of specialist species of typical physical environments have disappeared or decreased with the concomitant decline or extinction of their preferred niche within arable fields (i.e. species adapted to nutrient poor soils, either wet and sandy soil or calcareous rocky and dry soils). The difference of I_CS _in weed communities found in crop rotation and in monoculture is more related to the specialization of species to the crop species, and more specifically to the crop germination date. In our study, species that were specialist of specific crops could generally grow on a wide range of soil and climatic conditions, for example *Amaranthus retroflexus *and *Digitaria *sanguinalis which grow in maize crops.

## Conclusions

This study aimed to classify arable weeds along a generalist/specialist continuum and to assess if differences in disturbance patterns could lead to differences in the representation of generalist and specialist species in plant communities. Our study extends ecological approaches to an environment highly disturbed by human activities where it is not often easy to get clear expectations between contrasted and/or opposite forces that govern the assembly of community. The distinction of species into generalist or specialist species helps to highlight the general rules in the assembly of weed species into communities [39]. In our case, the classification of 152 arable weed species along a specialist/generalist gradient gives insights to their contrasted responses to changes in agriculture. We show that the relative proportion of generalist and specialist species is not constant but varies in relation to the frequency and intensity of disturbance that result from agricultural practices. Our results also show that, despite the high level of disturbance that characterises agroecosystems, ecological theories developed in more stable environments do apply and that either a sequence of disturbances of different nature (crop rotation) and/or the intensification of disturbances have favoured the most generalist species.

## Methods

### Weed flora and environmental data

We used plots data derived from Biovigilance Flore, a national monitoring scheme designed to survey changes in arable flora in relation to farming practices [40]. In the centre of 724 cultivated fields selected to cover the diversity of cultural techniques and environmental conditions occurring in annual crop fields throughout mainland France, comprehensive vegetation records were carried out in two separate 2000 m^2 ^plots, i.e. one sprayed with herbicides and a second located in an unsprayed control area. The 'herbicide' and the 'control' plots were randomly placed within the field, at least 20 m from the edge. The control plot is subjected to all cultivation practices but the herbicide treatments. Vegetation was recorded over the whole 2000 m^2^, twice a year, the first survey about a month after crop sowing and the second later on during crop development, i.e. early April for winter-sown crops and early July for spring and summer-sown crops. This sampling design provided a total of 2896 plots between 2002 and 2004. The abundance of each species was estimated using six abundance classes: '+' found once in the 2000 m^2 ^area; '1' less than 1 individual/m^2^; '2' 1-2 individual/m^2^; '3' 3-20 individuals/m^2^; '4' 21-50 individuals/m^2^; '5' more than 50 individuals/m^2 ^(for further sampling details, see [20]). In parallel to vegetation sampling, the monitoring scheme included the record of environmental variables as well as a survey describing agricultural practices collected by an interview with the farmers (Table 4). These were available for 694 out of the initial 724 fields. In this study, we extracted variables that are known to affect weed species distribution [19,20]. These included environmental variables such as altitude, climatic conditions (maximal and minimal temperature, total rainfall and evapotranspiration) derived from METEO-France climatic data with the AURELHY method of interpolation [41], soil texture (7 classes) and soil pH. Relevant agronomic variables were the crop rotation history, sowing date and tillage operations (type and number of operations, maximum depth of tillage). Precise data about herbicides, fertilizer levels and crop canopy were not available for all fields so are not included.

**Table 4:** Methods, references and data used to compute the six species habitat breadth indices and species niche position index

Method and reference	Applications to plant communities	# of plots	Habitat variables
**I_1 _(RS) **Reciprocal scaling [43]	[53]	2896 *	None
**I_2 _(CCA-SD) **Canonical Correspondence Analysis [44]	[54]	694	See list **
**I_3 _(CCA-Rao) **Canonical Correspondence Analysis [47] based on [45,46]	-	694	See list **
**I_4 _(OMI) **Outlying Mean Index analysis [48]	[55]	694	See list **
**I_5 _(IV) **IndVal [49]	[50]	694	See list **
**I_6 _(Sophy) **Species mean socio-ecological distances [51]	[51]	2896 *	None

2896 * = 724 fields with 2 plots surveyed twice a year

List ** includes habitat variables: Altitude, Mean temperature, Total rainfall, Evapotranspiration, Soil pH, Soil texture, Crop, Preceding crop, Sowing date, Tillage system and Tillage depth.

Tillage system: no-tillage (i.e. implementing direct drilling), minimum tillage which consists in only chiselling the soil and conventional tillage including tilling the soil with mouldboard plough followed by one or more harrow and/or cover-crops passage(s).

### Niche breadth indices

The degree of specialisation of individual species was computed using six different published methods, since none of them has received unanimous preference (Table 4) [42]. I_1 _and I_6 _were calculated solely on vegetation data and were estimated using the 2896 plot data. The other four indices were derived from both the environmental and agronomical variables described above and vegetation data which was pooled at the field level (n= 694). For indices I_2 _to I_5, _we pooled the floristic data of the herbicides plots and the unsprayed control plots as preliminary tests performed on each dataset independently indicated no significant differences in species ranking.

**Figure 5:**
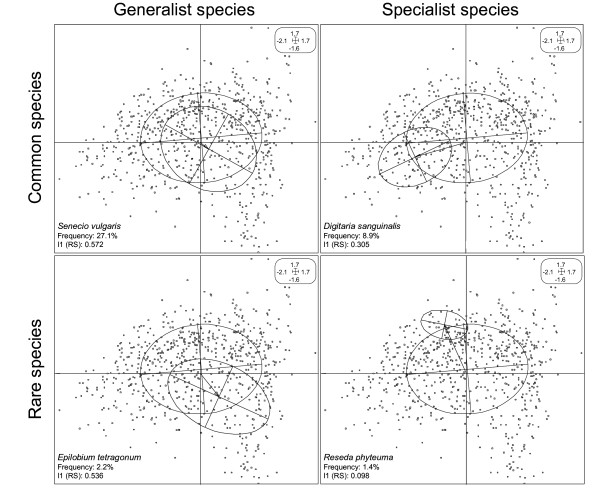
**Reciprocal scaling of species and plot records**. Each point represents a plot. Ellipses represent respective species habitat amplitudes (niche breadth) and deviances (niche position) compared to a theoretical species having a uniform distribution derived on the sampling (central ellipse). The centre of the ellipse is given by the mean of ordination scores, and axes of the ellipse are related to the variance of ordination scores. The figure gives an example of four contrasted species, each of the 152 species were assessed in this way. Both rare (Frequency < 5%) and common (Frequency > 5%) species can present a full range of responses from wide (generalist) to narrow (specialist) niche breadth.

Indices I_1 _to I_4 _use multivariate space to estimate niche breadth and relative position. In these analyses, a generalist species would cover a large volume of the n-dimensional ordination space, while a specialist species would be confined in a restricted area of that same ordination space (Fig. 5). Index I_1 _uses the method of 'Reciprocal scaling' which is based on the Correspondence Analysis of a species-sample matrix [43]. Indices I_2 _and I_3 _use Canonical Correspondence Analysis (CCA) as originally developed to discriminate species niches along environmental gradients. To measure the dispersion of the samples occupied by a species, Index I_2 _uses the standard deviation of species score in CCA [44] while Index I_3 _is based on the metrics of Rao [45,46] as proposed by Thioulouse *et al. *[47]. Indices I_1 _to I_3 _assume unimodal response curves of species to environmental gradients, while Index I_4_, the Outlying Mean Index (OMI) analysis makes no assumption about the shape of species response curves to the environment and, unlike CCA used in indices I_2 _and I_3_, Index I_4 _gives equal weight to species-rich and species-poor sites [48].

Index I_5 _uses the IndVal procedure [49] primarily designed to target indicator species according to ecological conditions using cluster analysis. It can also be used to distinguish generalist from specialist species [50], the most generalist species being associated with a broad partition while the most specialist species are associated with finer partitions i.e. clustered latter in the ordination tree.

Index I_6 _was based solely on the information given by species co-occurrence and assumed the principle that, all else being equal, generalists co-occur with many species across their range, while specialists co-occur with relatively few species [51]. Contrary to simple distance measures based on compositional dissimilarity, the distance between samples computed with index I_6 _is weighted by the ecological distance between the species with more weight given to species that are found under different conditions and less weight to species that are found under the same conditions, as detailed hereafter. First, the frequency of co-occurrence (F) between each pair of species was computed, where F(*a*/*b*) is the probability to find species *a *when species *b *is present (in general, F(*a*, *b*) is different from F(*b*, *a*)). The same kind of relationship can then be computed between a sample (S) and a species (*a*), the sample being considered as the mean of the species it contains:

(1) F(S,a)=[F(x1,a)+F(x2,a)+…+F(xi,a)+…+F(xn,a)]/n

with x1 to xn, n species present in the sample S. Hence, the distance between each pair of samples depends not only on their respective composition but also on a common global reference, i.e., the entire complement of species within the data set. Therefore even samples with no common species can be compared (i.e. their distance will not systematically equals 1) and the measure is independent of species richness in the samples.

### Classification of the French arable flora along a specialist-generalist gradient

For each index, we ranked all the species observed in at least ten fields from the most generalist (rank 1) to the most specialist species (rank n). Spearman's rank correlation test was then used to compare the classifications given by each index. For each species, we calculated the mean rank and standard deviation over the six indices. The mean rank resulted in a global index of specialization (I_S_); low I_S _indicated generalist species whereas high Is indicated specialist species. We cut the continuum of I_S _into three classes of equal size to classify species as either generalist, intermediate or specialist. The 25% species having the highest I_S _standard deviation were grouped into a fourth category called "varying category". Finally, we randomly rarefied 5 species (*Lolium multiflorum*, *Raphanus raphanistrum*, *Senecio vulgaris*, *Stellaria media *and *Veronica persica*) to 75%, 50% and 25% of their real occurrences in order to check for a frequency-dependence bias in our classification.

Finally, we computed the frequency and abundance of each species. The frequency is the number of occurrences where the species is present divided by the total number of surveyed plots while the mean abundance is given by the following formula:

Mean abundance=11.5*n3+35.5*n4+75.5*n5+1.5(N–n3–n4–n5)/N

with n3, n4, n5 the number of samples within the coefficients classes 3, 4 and 5 respectively and N, the total number of samples. 11.5, 35.5 and 75.5 correspond to the mean plant * m^-2 ^density of the abundance coefficient classes 3, 4 and 5, respectively [52].

### Community response to disturbance regimes in agricultural systems

The average degree of specialization of a given community I_CS _was calculated as the mean I_S _of the species present. Species belonging to the 'varying category' were not used in the I_CS _calculations. As differences in I_CS _between communities could either reflect (i) a difference in the proportion of generalist species in the community, (ii) a difference in the proportion of specialists or (iii) a combination of both, we used the proportion of specialist, intermediate and generalist species in the community as additional indicators.

The comparison of weed communities in monoculture versus crop rotation was based on data from the Biovigilance Flore dataset. We used vegetation plots sampled in maize cultivated in monoculture for at least the last four years (n = 235 fields) and vegetation plots recorded in maize fields cropped within a crop-rotation just after winter wheat (n = 169 fields). We compared I_CS _in monoculture and crop rotation using a Wilcoxon test and the proportion of generalist, intermediate and specialist species using a χ^2^-test. To ensure that the influence of soil type on the I_CS _index is not confounding the analysis of the cropping systems, we have analysed the distribution of cropping systems as well as the distribution of I_CS _values across soil types.

The comparison of weed communities occurring in the 1970 s and in the 2000 s was carried out by using a repeat survey (2005-2006) of 158 fields initially surveyed between 1968 and 1976 (for further sampling details, see [38]). I_CS _and the proportion of species belonging to the three classes were compared as described above.

## Authors' contributions

GF collected the data, carried out the analysis and drafted the manuscript. XR was at the initiative of the project and supervised its development and funding. SP helped develop the theoretical background and assisted in drafting and revised the manuscript. All authors read and approved the final manuscript.

## Supplementary Material

Additional file 1 **Classification of arable weed species according to their niche breadth**. Additional file descriptions text (including details of how to view the file, if it is in a non-standard format). The file is in PDF format. It gives a table with the mean degree of specialisation (I_S_) of 152 weed species according to the score given by six different niche breadth indices that are also given in the table. Click here for file
