# Supplementary material for: A specialist-generalist classification of the arable flora and its response to changes in agricultural practices
Source: BMC Ecol. 2010 Sep 1;10:20. doi: 10.1186/1472-6785-10-20 (PMC2939635; doi:10.1186/1472-6785-10-20)
Supplement: Additional file 1 — Classification of arable weed species according to their niche breadth. Additional file descriptions text (including details of how to view the file, if it is in a non-standard format). The file is in PDF format. It gives a table with the mean degree of specialisation (IS) of 152 weed species according to the score given by six different niche breadth indices that are also given in the table. [file 1472-6785-10-20-S1.PDF]

Additional file 1: Classification of arable weed species according to their niche breadth (based on six different indexes: I_1 to I_6 and a global averaged index: IS). Frequency was calculated as the proportion of samples in which a species occurred according to 2896 samples recorded throughout France. Geographical range was calculated as the sum of pairwise distances between all the samples in which a species was present.

Abbreviations refer to the published method used to quantify niche breadth. RS: Reciprocal scaling, OMI: Outlying Mean Index, CCA-SD: Canonical Correspondence Analysis using Standard Deviation, CCA-Rao: Canonical Correspondence Analysis using Rao's indice of diversity, IndVal: Indicator Values Procedure, Sophy: ecological distances between species.

The most generalist weed species	I1 (RS)	I2 (OMI)	I3 (CCA-SD)	I4 (CCA-Rao)	I5 (IndVal)	I6 (Sophy)	Freq.	Geogr. Range	IS Mean Indices Rank	Stand. Dev.
Stellaria media (L.) Vill.	0.594	2.529	0.517	1.263	1.000	0.311	0.247	1.525E+11	14.833	13.631
Lolium multiflorum Lam.	0.516	2.784	0.520	1.251	1.000	0.254	0.203	1.494E+11	15.667	10.483
Veronica persica Poir.	0.552	2.664	0.518	1.312	1.000	0.237	0.186	1.652E+11	16.667	15.019
Raphanus raphanistrum L.	0.543	2.476	0.528	1.251	1.000	0.277	0.091	1.146E+11	17.500	16.101
Senecio vulgaris L.	0.572	2.457	0.518	1.205	1.000	0.297	0.271	1.353E+11	19.167	16.191
Elytrigia repens (L.) Desv. ex Nevski	0.530	2.316	0.521	1.273	1.000	0.277	0.051	1.984E+11	22.000	22.361
Veronica arvensis L.	0.431	2.624	0.522	1.206	1.000	0.285	0.043	1.628E+11	22.500	15.997
Lapsana communis L.	0.551	2.559	0.520	1.237	1.000	0.205	0.062	8.291E+10	23.500	22.845
Poa annua L.	0.540	2.431	0.504	1.196	1.000	0.254	0.211	1.261E+11	25.167	16.984
Rumex acetosa L.	0.552	2.466	0.555	1.282	0.250	0.236	0.012	6.971E+10	26.000	20.833
Capsella bursa-pastoris (L.) Medik.	0.503	2.515	0.505	1.220	1.000	0.226	0.159	1.332E+11	26.500	15.798
Lamium purpureum L.	0.503	2.435	0.510	1.244	1.000	0.228	0.081	1.330E+11	27.167	18.515
Viola arvensis Murray	0.523	2.630	0.501	1.072	1.000	0.218	0.229	1.571E+11	28.833	16.557
Sonchus oleraceus L.	0.523	2.383	0.514	1.273	0.250	0.306	0.074	1.440E+11	29.000	18.868
Cirsium arvense (L.) Scop.	0.431	2.849	0.492	1.078	1.000	0.214	0.158	1.873E+11	32.333	20.621
Euphorbia helioscopia L.	0.492	2.685	0.503	1.087	1.000	0.177	0.079	1.603E+11	33.667	24.709
Taraxacum campylodes G.E.Haglund	0.479	2.732	0.510	1.178	0.091	0.291	0.079	1.212E+11	34.833	23.738
Rumex crispus L.	0.521	2.447	0.519	1.242	0.091	0.292	0.043	1.895E+11	35.000	26.696
Arrhenatherum elatius subsp. *bulbosum* (Willd.) Schübler & G.Martens	0.475	2.541	0.519	1.183	0.091	0.294	0.012	1.109E+11	36.667	24.102
Matricaria recutita L.	0.432	2.762	0.508	1.023	0.500	0.206	0.151	1.101E+11	38.000	18.009
Sinapis arvensis L.	0.422	2.781	0.489	1.020	0.500	0.215	0.161	1.354E+11	39.667	15.702
Daucus carota L.	0.375	2.746	0.490	1.027	1.000	0.214	0.050	1.377E+11	39.667	23.655
Ranunculus sardous Crantz	0.436	2.407	0.483	1.341	0.250	0.218	0.041	6.952E+10	41.833	17.535
Avena fatua L.	0.397	2.694	0.495	0.969	1.000	0.183	0.057	1.932E+11	43.500	26.431
Anagallis arvensis L.	0.403	2.523	0.490	1.089	1.000	0.171	0.152	1.635E+11	44.333	27.602
Geranium dissectum L.	0.416	2.749	0.500	0.925	0.500	0.191	0.083	8.871E+10	44.833	22.026
Lactuca serriola L.	0.348	2.497	0.497	1.142	0.167	0.254	0.029	1.082E+11	47.167	19.591
Polygonum aviculare L.	0.423	2.461	0.486	1.028	1.000	0.170	0.280	1.813E+11	48.000	26.820
Fallopia convolvulus (L.) Á.Löve	0.349	2.780	0.489	1.052	1.000	0.153	0.195	1.922E+11	48.000	36.097
Anthemis arvensis L.	0.440	2.330	0.474	0.949	0.167	0.315	0.027	1.459E+11	48.333	16.908
Matricaria perforata Mérat	0.541	2.393	0.501	1.121	0.111	0.169	0.062	1.232E+11	49.167	27.721
Ranunculus arvensis L.	0.512	2.362	0.480	1.007	0.111	0.244	0.040	1.305E+11	49.333	15.553
Rumex obtusifolius L.	0.440	2.255	0.464	1.129	0.111	0.304	0.066	1.111E+11	50.000	19.570
Matricaria discoidea DC.	0.548	2.766	0.444	0.803	0.200	0.207	0.016	3.727E+10	50.667	29.210
Chenopodium album L.	0.464	2.210	0.457	0.856	1.000	0.244	0.435	1.923E+11	51.000	28.255
Galium aparine L.	0.419	2.710	0.472	0.920	0.333	0.186	0.248	1.413E+11	52.333	18.227
Plantago lanceolata L.	0.332	2.940	0.489	1.161	0.091	0.210	0.022	1.359E+11	52.667	32.133
Alopecurus myosuroides Huds.	0.338	2.861	0.463	0.835	0.500	0.221	0.206	1.313E+11	53.000	29.360
Minimum	0.332	2.210	0.444	0.803	0.091	0.153	0.012	3.73E+10	14.833	10.483
1st Quartile	0.422	2.438	0.489	1.024	0.213	0.206	0.045	1.16E+11	26.125	16.645
Median	0.477	2.535	0.501	1.135	1.000	0.227	0.080	1.36E+11	37.333	20.106
3rd Quartile	0.528	2.743	0.518	1.241	1.000	0.277	0.193	1.62E+11	48.000	26.000
Maximum	0.594	2.940	0.555	1.341	1.000	0.315	0.435	1.98E+11	53.000	36.097

List continued

Intermediate weed species	I1 (RS)	I2 (OMI)	I3 (CCA-SD)	I4 (CCA-Rao)	I5 (IndVal)	I6 (Sophy)	Freq.	Geogr. Range	IS Mean Indices Rank	Stand. Dev.
Oxalis fontana Bunge	0.421	2.231	0.464	1.106	0.111	0.272	0.022	6.932E+10	54.833	17.082
Fumaria officinalis L.	0.356	2.669	0.476	1.035	0.500	0.165	0.135	1.719E+11	55.167	26.529
Vicia sativa L.	0.497	2.098	0.493	1.084	0.111	0.197	0.032	7.543E+10	56.000	24.893
Cerastium glomeratum Thuill.	0.429	2.194	0.487	1.154	0.111	0.207	0.062	8.434E+10	56.000	18.592
Solanum tuberosum L.	0.505	2.339	0.498	1.163	0.083	0.169	0.009	1.325E+11	57.167	32.850
Sonchus asper (L.) Hill	0.363	2.438	0.466	0.884	1.000	0.184	0.181	1.332E+11	57.167	26.586
Torilis arvensis (Huds.) Link	0.577	2.108	0.468	1.060	0.091	0.212	0.006	1.167E+11	58.167	29.802
Centaurea cyanus L.	0.393	2.599	0.471	0.909	0.059	0.360	0.011	1.248E+11	58.333	33.861
Brassica nigra (L.) W.D.J.Koch	0.335	2.586	0.437	0.867	0.167	0.308	0.011	1.174E+11	60.500	27.238
Picris echioides L.	0.320	2.728	0.444	0.875	0.500	0.193	0.021	1.130E+11	61.833	28.428
Papaver rhoeas L.	0.306	2.732	0.470	0.875	0.500	0.173	0.155	1.598E+11	61.833	31.254
Bidens tripartita L.	0.484	2.119	0.431	0.885	0.091	0.317	0.007	9.474E+10	63.833	30.113
Brassica napus L.	0.320	2.490	0.483	1.077	0.059	0.241	0.066	1.167E+11	65.167	31.206
Polygonum persicaria L.	0.372	2.141	0.460	0.833	1.000	0.176	0.198	1.623E+11	66.167	30.058
Veronica hederifolia L.	0.436	2.634	0.426	0.805	0.167	0.165	0.186	1.527E+11	68.333	26.323
Arabidopsis thaliana (L.) Heynh.	0.548	2.202	0.431	1.044	0.091	0.156	0.022	8.556E+10	69.667	32.009
Solanum nigrum L.	0.389	2.168	0.439	0.808	1.000	0.165	0.254	1.834E+11	70.667	32.500
Convolvulus arvensis L.	0.351	2.217	0.465	0.828	0.333	0.172	0.161	2.051E+11	71.833	19.403
Poa trivialis L.	0.372	2.033	0.474	1.020	0.111	0.179	0.021	1.024E+11	72.500	17.260
Juncus bufonius L.	0.374	2.298	0.483	0.941	0.050	0.221	0.032	9.569E+10	72.833	31.307
Agrostis stolonifera L.	0.366	2.084	0.441	0.914	0.063	0.313	0.024	1.343E+11	75.167	30.030
Dactylis glomerata L.	0.304	2.442	0.497	1.036	0.091	0.105	0.008	6.937E+10	75.167	34.007
Triticum aestivum L.	0.354	2.502	0.470	0.790	0.053	0.214	0.036	8.501E+10	76.833	26.667
Mercurialis annua L.	0.344	2.242	0.417	0.754	0.333	0.203	0.203	2.288E+11	77.833	22.952
Trifolium pratense L.	0.324	2.180	0.446	0.977	0.111	0.176	0.006	1.831E+11	78.000	12.819
Rapistrum rugosum (L.) All.	0.397	2.118	0.443	0.783	0.083	0.236	0.007	5.965E+10	79.000	19.740
Cynodon dactylon (L.) Pers.	0.433	1.791	0.411	0.648	0.125	0.265	0.059	9.282E+10	80.167	35.494
Polygonum lapathifolium L.	0.378	2.220	0.442	0.950	0.063	0.183	0.056	1.882E+11	80.167	15.225
Lamium amplexicaule L.	0.351	2.245	0.440	0.980	0.091	0.143	0.016	1.432E+11	82.833	15.181
Equisetum arvense L.	0.248	2.112	0.419	0.776	0.333	0.204	0.055	2.745E+11	85.167	28.597
Artemisia vulgaris L.	0.255	2.397	0.421	0.748	0.333	0.165	0.018	1.356E+11	85.833	30.207
Calystegia sepium (L.) R.Br.	0.381	1.733	0.404	0.723	0.333	0.189	0.100	2.238E+11	87.000	31.969
Anthemis cotula L.	0.302	2.564	0.446	0.748	0.100	0.129	0.010	1.197E+11	87.500	26.254
Aethusa cynapium L.	0.368	2.660	0.407	0.784	0.059	0.141	0.051	1.503E+11	88.667	29.375
Setaria viridis (L.) P.Beauv.	0.313	2.117	0.423	0.640	0.500	0.140	0.043	1.380E+11	90.000	30.490
Rumex acetosella L.	0.311	1.919	0.439	0.871	0.111	0.173	0.012	2.476E+10	90.000	16.862
Atriplex patula L.	0.266	2.344	0.416	0.633	0.333	0.129	0.090	1.434E+11	91.333	31.883
Panicum miliaceum L.	0.341	1.935	0.411	0.633	0.333	0.168	0.025	2.298E+11	92.333	28.235
Minimum	0.248	1.733	0.404	0.633	0.050	0.105	0.006	2.48E+10	54.833	12.819
1st Quartile	0.321	2.117	0.427	0.783	0.091	0.165	0.013	9.50E+10	61.833	23.437
Median	0.365	2.226	0.443	0.875	0.111	0.181	0.032	1.33E+11	72.667	28.513
3rd Quartile	0.396	2.478	0.470	1.010	0.333	0.214	0.084	1.62E+11	82.167	31.242
Maximum	0.577	2.732	0.498	1.163	1.000	0.360	0.254	2.75E+11	92.333	35.494

List continued

The most specialist weed species	I1 (RS)	I2 (OMI)	I3 (CCA-SD)	I4 (CCA-Rao)	I5 (IndVal)	I6 (Sophy)	Freq.	Geogr. Range	IS Mean Indices Rank	Stand. Dev.
Euphorbia peplus L.	0.361	2.679	0.321	0.765	0.083	0.140	0.005	2.115E+11	92.667	34.038
Setaria pumila (Poir.) Roem. & Schult.	0.337	2.186	0.446	0.858	0.063	0.103	0.029	2.070E+11	93.833	18.059
Galeopsis tetrahit L.	0.447	2.105	0.388	0.727	0.091	0.112	0.011	6.891E+10	94.000	26.994
Sonchus arvensis L.	0.307	2.100	0.448	0.698	0.053	0.215	0.032	1.442E+11	94.167	22.807
Aphanes arvensis L.	0.413	2.272	0.405	0.592	0.091	0.093	0.071	7.538E+10	95.667	25.296
Amaranthus retroflexus L.	0.334	1.916	0.393	0.622	0.333	0.145	0.171	1.564E+11	96.833	29.796
Echinochloa crus-galli (L.) P.Beauv.	0.326	1.801	0.401	0.606	0.333	0.151	0.187	1.856E+11	98.000	30.616
Chenopodium polyspermum L.	0.316	2.009	0.394	0.792	0.091	0.122	0.042	1.919E+11	102.667	11.528
Zea mays L.	0.427	1.991	0.374	0.560	0.053	0.176	0.005	4.250E+10	103.667	26.849
Digitaria sanguinalis (L.) Scop.	0.305	1.700	0.383	0.577	0.111	0.148	0.089	1.032E+11	109.500	20.751
Geranium pusillum L.	0.198	2.034	0.474	0.763	0.048	0.089	0.004	3.338E+10	110.333	30.231
Kickxia elatine (L.) Dumort.	0.330	2.010	0.372	0.694	0.091	0.068	0.020	9.604E+10	110.833	18.179
Plantago major L.	0.180	2.379	0.378	0.451	0.111	0.066	0.037	1.140E+11	111.333	33.119
Trifolium repens L.	0.257	1.827	0.360	0.694	0.091	0.164	0.010	1.152E+11	112.500	15.097
Phalaris paradoxa L.	0.285	1.815	0.323	0.600	0.059	0.195	0.006	6.737E+10	114.500	19.788
Lithospermum arvense L.	0.173	1.958	0.337	0.510	0.125	0.145	0.004	7.548E+10	115.000	27.006
Misopates orontium (L.) Raf.	0.214	2.143	0.341	0.699	0.091	0.052	0.006	5.813E+10	115.167	23.140
Legousia speculum-veneris (L.) Chaix	0.182	1.751	0.393	0.620	0.167	0.063	0.006	2.386E+10	116.167	30.400
Kickxia spuria (L.) Dumort.	0.180	2.003	0.382	0.468	0.125	0.083	0.057	1.340E+11	116.167	27.107
Verbena officinalis L.	0.205	2.003	0.347	0.450	0.125	0.094	0.011	8.201E+10	116.500	26.669
Datura stramonium L.	0.270	1.561	0.371	0.503	0.111	0.114	0.046	9.296E+10	117.500	23.056
Chenopodium hybridum L.	0.273	2.067	0.417	0.564	0.045	0.096	0.022	9.805E+10	119.333	16.790
Panicum dichotomiflorum Michx.	0.209	1.977	0.319	0.497	0.111	0.085	0.008	1.139E+11	119.333	24.452
Ambrosia artemisiifolia L.	0.279	1.769	0.430	0.571	0.053	0.080	0.015	6.113E+10	120.500	14.221
Paspalum dilatatum Poir.	0.279	1.418	0.293	0.331	0.063	0.202	0.008	1.161E+10	121.500	26.563
Veronica polita Fr.	0.176	2.399	0.384	0.484	0.048	0.065	0.010	4.813E+10	122.833	29.863
Setaria verticillata (L.) P.Beauv.	0.192	1.543	0.346	0.438	0.125	0.087	0.039	1.694E+11	123.167	28.363
Amaranthus blitoides S.Watson	0.220	1.836	0.314	0.384	0.111	0.069	0.008	1.268E+11	123.333	25.636
Digitaria ischaemum (Schreb.) Mühl.	0.212	1.523	0.311	0.549	0.111	0.035	0.004	1.364E+11	126.167	27.193
Conyza canadensis (L.) Cronquist	0.205	1.913	0.373	0.451	0.053	0.120	0.009	1.017E+11	126.333	5.497
Phytolacca americana L.	0.213	1.170	0.299	0.220	0.111	0.064	0.009	5.425E+10	130.167	28.574
Xanthium strumarium L.	0.291	1.727	0.273	0.370	0.077	0.075	0.011	3.774E+10	130.333	14.648
Gnaphalium uliginosum L.	0.113	1.917	0.272	0.273	0.091	0.084	0.010	5.010E+10	130.333	21.838
Chaenorrhinum minus (L.) Lange	0.078	1.674	0.292	0.285	0.125	0.029	0.008	1.020E+11	131.500	32.402
Amaranthus albus L.	0.094	1.164	0.287	0.517	0.053	0.101	0.006	1.185E+11	137.500	10.658
Reseda phyteuma L.	0.098	1.149	0.183	0.253	0.077	0.033	0.013	1.138E+11	142.500	15.457
Stachys arvensis (L.) L.	0.041	0.572	0.137	0.174	0.063	0.071	0.005	1.363E+11	142.833	15.302
Arenaria serpyllifolia L.	0.170	1.522	0.077	0.201	0.048	0.080	0.007	6.097E+10	144.833	5.014
Minimum	0.041	0.572	0.077	0.174	0.045	0.029	0.004	1.16E+10	92.667	5.014
1st Quartile	0.181	1.681	0.311	0.441	0.060	0.069	0.006	6.10E+10	109.708	17.107
Median	0.217	1.915	0.366	0.533	0.091	0.091	0.010	9.99E+10	116.333	24.874
3rd Quartile	0.307	2.028	0.391	0.621	0.111	0.136	0.031	1.32E+11	125.458	28.071
Maximum	0.447	2.679	0.474	0.858	0.333	0.215	0.187	2.11E+11	144.833	34.038

List continued

Varying weed species	I1 (RS)	I2 (OMI)	I3 (CCA-SD)	I4 (CCA-Rao)	I5 (IndVal)	I6 (Sophy)	Freq.	Geogr. Range	IS Mean Indices Rank	Stand. Dev.
Rather classified among the most generalist										
Portulaca oleracea L.	0.667	2.053	0.523	1.469	0.250	0.560	0.033	5.986E+10	26.167	37.267
Geranium rotundifolium L.	0.360	2.893	0.542	1.247	0.059	0.330	0.033	1.081E+11	38.500	44.033
Veronica agrestis L.	0.558	2.880	0.474	1.421	0.063	0.215	0.007	1.503E+11	39.000	38.531
Bromus hordeaceus L.	0.492	2.316	0.519	1.511	0.053	0.365	0.006	1.632E+11	40.667	44.338
Valerianella locusta (L.) Laterr.	0.584	2.861	0.433	1.041	0.053	0.308	0.011	7.220E+10	48.667	45.006
Epilobium tetragonum L.	0.536	2.303	0.484	1.154	0.050	0.376	0.022	1.350E+11	50.333	44.705
Ranunculus repens L.	0.465	2.038	0.477	1.169	0.063	0.320	0.016	1.251E+11	55.667	37.263
Rather classified as intermediary										
Hordeum vulgare L.	0.320	2.840	0.516	0.763	0.083	0.245	0.023	5.358E+10	61.167	39.671
Achillea millefolium L.	0.494	1.769	0.411	1.037	0.111	0.489	0.005	1.064E+11	63.333	43.450
Holcus mollis L.	0.297	2.574	0.520	1.053	0.111	0.137	0.006	4.185E+10	63.667	38.556
Helianthus annuus L.	0.541	1.884	0.412	0.824	0.167	0.324	0.029	9.055E+10	64.500	42.327
Carex hirta L.	0.430	1.970	0.443	1.292	0.063	0.243	0.020	1.207E+11	66.833	38.542
Beta vulgaris L.	0.333	3.078	0.439	0.873	0.043	0.479	0.008	1.292E+11	67.667	51.610
Spergula arvensis L.	0.454	1.970	0.472	1.137	0.045	0.268	0.017	8.880E+10	69.500	44.992
Trifolium arvense L.	0.308	2.536	0.550	0.995	0.053	0.174	0.012	9.872E+10	69.500	41.452
Potentilla reptans L.	0.446	2.086	0.450	0.857	0.053	0.357	0.012	8.937E+10	70.333	38.012
Chenopodium murale L.	0.501	2.020	0.454	0.782	0.053	0.417	0.004	1.024E+11	70.667	42.999
Picris hieracioides L.	0.268	3.174	0.461	0.926	0.167	0.118	0.017	7.006E+10	70.833	41.229
Erodium cicutarium (L.) L'Hér.	0.396	1.656	0.480	0.992	0.045	0.355	0.009	5.292E+10	77.000	49.555
Myosotis arvensis Hill	0.509	2.416	0.451	0.894	0.050	0.150	0.059	9.453E+10	77.167	36.644
Sherardia arvensis L.	0.167	2.276	0.503	0.815	0.333	0.142	0.014	1.443E+11	78.000	41.166
Vicia hirsuta (L.) Gray	0.354	1.321	0.485	1.290	0.048	0.194	0.010	1.012E+11	82.167	49.965
Malva sylvestris L.	0.261	2.205	0.397	0.664	0.500	0.231	0.010	1.484E+11	82.333	36.119
Scandix pecten-veneris L.	0.402	1.211	0.424	1.221	0.048	0.251	0.009	6.991E+10	83.500	49.436
Ammi majus L.	0.360	1.961	0.322	0.631	0.250	0.343	0.018	2.427E+11	83.833	43.971
Silene latifolia subsp. *alba* (Mill.) Greuter & Burdet	0.236	2.855	0.443	0.708	0.083	0.129	0.015	1.264E+11	90.000	38.601
Bromus sterilis L.	0.293	2.728	0.390	0.589	0.059	0.217	0.022	1.457E+11	90.667	37.646
Rather classified among the most specialist										
Erophila verna (L.) Chevall.	0.373	1.697	0.391	1.087	0.050	0.218	0.003	5.244E+10	91.167	40.321
Sorghum halepense (L.) Pers.	0.465	1.098	0.374	0.766	0.053	0.284	0.011	8.413E+10	93.000	46.694
Rubus spp.	0.389	1.635	0.368	0.542	0.333	0.172	0.023	1.528E+11	97.333	38.073
Apera spica-venti (L.) P.Beauv.	0.454	2.817	0.351	0.384	0.043	0.129	0.023	4.703E+10	98.000	52.564
Medicago lupulina L.	0.183	3.008	0.388	0.772	0.059	0.089	0.003	9.379E+10	100.167	44.927
Thlaspi arvense L.	0.155	1.888	0.427	0.842	0.333	0.087	0.007	1.502E+11	100.667	37.371
Amaranthus hybridus L.	0.409	1.534	0.368	0.416	0.053	0.274	0.004	7.481E+10	103.833	43.118
Euphorbia exigua L.	0.142	2.011	0.178	0.211	0.333	0.215	0.009	4.227E+10	106.833	46.760
Stachys annua (L.) L.	0.11	2.21	0.445	0.36	0.13	0.04	0.01	6.795E+10	109.500	38.025
Anagallis foemina Mill.	0.132	3.012	0.371	0.532	0.059	0.062	0.012	1.688E+11	111.500	49.628
Glebionis segetum (L.) Fourr.	0.290	1.244	0.225	0.378	0.125	0.203	0.006	1.148E+11	113.167	36.288
Minimum	0.105	1.098	0.178	0.211	0.043	0.044	0.003	4.19E+10	39.000	36.119
1st Quartile	0.285	1.855	0.390	0.656	0.052	0.148	0.007	7.17E+10	66.250	38.417
Median	0.381	2.145	0.441	0.849	0.059	0.224	0.011	1.00E+11	77.583	41.889
3rd Quartile	0.465	2.750	0.475	1.061	0.125	0.321	0.018	1.37E+11	94.083	44.995
Maximum	0.584	3.174	0.550	1.511	0.500	0.489	0.059	2.43E+11	113.167	52.564
